# Examining the predictive validity of a managerial coaching scale: a longitudinal study

**DOI:** 10.3389/fpsyg.2024.1277422

**Published:** 2024-04-02

**Authors:** Katie Stone, Kim Nimon, Andrea D. Ellinger

**Affiliations:** ^1^Director of Student Wellness and Professional Formation, School of Medicine, The University of Texas at Tyler, Tyler, TX, United States; ^2^Department of Human Resource Development, Soules College of Business, The University of Texas at Tyler, Tyler, TX, United States

**Keywords:** managerial coaching, longitudinal study, employee attitudes, role clarity, job satisfaction, organization commitment

## Abstract

Managerial coaching remains a widespread and popular organizational development intervention applied across numerous industries to enhance critical workplace outcomes and employee attitudes, yet no studies to date have evaluated the temporal precedence within these relationships. This study sought to assess the predictive validity of the widely used Employee Perceptions of Supervisor/Line Manager Coaching Behavior Measure managerial coaching scale (CBI), employing a longitudinal design and following the testing of the causal hypothesized relationship framework. Three hypotheses were evaluated using three commonly associated variables with managerial coaching (role clarity, job satisfaction, and organization commitment), using longitudinal data collected over two waves from full-time US employees (*n* = 313). The study followed a two-wave design, collecting data over two time points to test for longitudinal measurement invariance and three reciprocal cross-lagged models. Results detected statistically significant cross-lagged and reciprocal cross-lagged effects in the role clarity and organization commitment models, highlighting a reciprocal relationship between managerial coaching behaviors and the two variables. However, only the reciprocal cross-lagged effect was statistically significant in the job satisfaction model. Findings suggest the predictive validity of the CBI scale for role clarity and organization commitment. Moreover, results indicate employee attitudes influenced managerial coaching behaviors over time across all three models, emphasizing the potential impact of employee attitudes on leadership effectiveness. This study highlights the complex relationships between managerial coaching and workplace outcomes, offering nuanced insights for improved understanding.

## 1 Introduction

Coaching has become such a popular and prevalent developmental intervention that the International Coach Federation (ICF) estimated that coaching professionals generated an approximated annual revenue of $4.564 billion (US) in 2022, with an estimated number of 109,200 coach practitioners worldwide (Global Coaching Study, 2023). Coaching is “recognized as a powerful vehicle for increasing performance, achieving results and optimizing personal effectiveness” (Bachkirova et al., 2024, p. 1). According to Bachkirova et al. (2024), coaching is “a human development process that involves structured, focused interaction and the use of appropriate strategies, tools and techniques to promote desirable and sustainable change for the benefit of the client and potentially for other stakeholders” (p. 1). One particular type or genre of coaching in the context of work is managerial coaching (Ellinger et al., 2024). Managerial coaching is considered to be a developmental-focused process facilitated by a manager who encourages the growth and learning of their employees (Ellinger et al., 2003; Beattie et al., 2014; Cox et al., 2024). More specifically Ellinger et al. (2024) have defined managerial coaching as “a manager or supervisor serving as a coach or facilitator of learning in the workplace setting, in which they enact specific behaviors that enable their employees (coachees) to learn and develop” (p. 264). The proliferation of managerial coaching as a business practice in organizations has occurred as a consequence of managers being held more accountable and responsible for employee development (Hagen, 2012). Moreover, employees have also come to expect more collaborative, developmental, and motivational supervisory styles (Hagen, 2012; Lawrence, 2017).

Current research has positively linked managerial coaching with numerous organizational and individual benefits, including increased employee job performance (Kim et al., 2013; Kim, 2014; Kim and Kuo, 2015; Pousa et al., 2018), employee work satisfaction (Ellinger et al., 2003; Kalkavan and Katrinli, 2014; Kim et al., 2014; Ali et al., 2018), and increased financial goal attainment (Dahling et al., 2016). However, despite the increased and widespread application of managerial coaching, most evidence regarding its effectiveness lies predominately in the practitioner literature (Kim, 2014). Most scholars agree that managerial coaching provides numerous advantages, but many aspects of managerial coaching are not yet fully explored. Lawrence (2017) advocated for advancing managerial coaching research through longitudinal studies to understand the effect of time on the relationships between managerial coaching and work-related outcomes. Currently, very few published longitudinal studies on managerial coaching exist, despite numerous calls in the literature base for conducting such studies (Beattie et al., 2014; Dahling et al., 2016; Lawrence, 2017; Ratiu et al., 2017; McCarthy and Milner, 2020). Among the few, such longitudinal studies incorporated a sequential design thereby hindering the ability to fully understand the temporal relationship between managerial coaching and the dependent variables (Preacher, 2015). The current lack of longitudinal studies within the managerial coaching literature indicates a need for increased study design rigor since cross-sectional data may lead to inflated effect sizes and biased conclusions (Cole and Maxwell, 2003). Although longitudinal studies are not a cure-all for methodological issues associated with cross-sectional studies, they do offer additional insights into how variables influence and change over time (Hsiao, 2007; Stritch, 2017).

At present, more than 10 scales are available to assess and measure managerial coaching behaviors and skills, but only a few are commonly used within the literature (Hagen and Peterson, 2015; Lawrence, 2017). One of the more widely known and utilized coaching scales is the Employee Perceptions of Supervisor/Line Manager Coaching Behavior measure (CBI; Ellinger et al., 2003). The CBI, however, has been predominately used in cross-sectional research studies, limiting understanding of the predictive validity of the scale (Hagen and Park, 2013; Buljac-Samardzic and van Woerkom, 2015). Thus, more research is needed that examines the psychometric properties of this scale, along with studies that extend beyond cross-sectional designs and employ longitudinal approaches.

Given the booming coaching industry, managers and organizations that utilize managerial coaching as a business practice need to fully understand all aspects that can affect coaching effectiveness by incorporating robust measurement instruments and longitudinal designs to further understand causal relationships between managerial coaching and work performance and attitudinal outcomes over time. Therefore, the purpose of this study was to assess the predictive validity of the Employee Perceptions of Supervisor/Line Manager Coaching Behavior Measure (CBI) by determining measurement invariance and then conducting a cross-lagged panel design. The managerial coaching outcomes of interest that emerged from an extensive literature search include role clarity, job satisfaction, and organization commitment. Current managerial coaching literature has relied upon predominately cross-sectional designs to explain and infer relationships among variables, limiting understanding and leading to incorrect assumptions (Mitchell and Maxwell, 2013). In contrast, this study employed a robust longitudinal design to promote better understanding of managerial coaching’s influence on role clarity, job satisfaction, and organizational commitment over time, focusing insights on a widely used managerial coaching scale (CBI). The sections that follow describe the theoretical framing of this study and elaborate on the methods and analyses undertaken. The findings are presented along with implications for practice and future research.

## 2 Theoretical background and research hypotheses

### 2.1 Determining the dependent variables

A comprehensive literature review was conducted via Google Scholar and an institutional search engine to determine the dependent variables for the present study. A total of 374 quantitative studies that used the Ellinger et al. (2003) scale as an independent variable were identified to form a comprehensive list of dependent variables and verify strong statistically significant cross-sectional relationships between managerial coaching and each dependent variable. Verifying a cross-sectional relationship is a necessary precursor to a longitudinal study design (Kenny, 1975). The 374 identified article abstracts were reviewed and narrowed down to 23 studies. Articles were removed based on the following conditions: if they were duplicates, if they did not use the Ellinger et al. (2003) CBI scale, if managerial coaching was not modeled as an independent variable, if the article was conceptual or used a qualitative research design, if the article did not assess work-related psychological or performance outcomes, or if they were dissertations or not written in English. Within the 23 results identified, the top five most incorporated dependent variables were: job performance (12 studies), job satisfaction (seven studies), organizational commitment (five studies), role clarity (four studies), and customer orientation (three studies). Job performance was the most commonly utilized variable in managerial coaching studies that used the CBI scale. However, further examination of the job performance variable revealed potential difficulties. Numerous studies that incorporated job performance as a dependent variable had mixed results. They either demonstrated no association with managerial coaching (Kim et al., 2013; Kim, 2014) or reported a negative association with managerial coaching (Kim et al., 2014). A more recent study that incorporated job performance as a dependent variable while using the CBI scale as the independent variable, collected job performance reviews as their measurement, which proved impossible to replicate given the present study’s sample frame (Hsu et al., 2019). As a result, job performance was excluded from this study. However, given the widespread use of role clarity, job satisfaction, and organization commitment with the Ellinger et al. (2003) managerial coaching scale, these three dependent variables were chosen.

Managerial coaching has been both conceptually and empirically related to the aforementioned selected dependent variables in extant research. Described as “a state of employee mind-set about their job,” role clarity plays a significant role in organizational effectiveness (Kim et al., 2014, p. 241; Rizzo et al., 1970). Classic managerial coaching behaviors such as developing employees, providing structure, and supervisory communication have been connected to increased role clarity (Rizzo et al., 1970; Ellinger et al., 2003). Satisfaction with work is defined as an employee’s emotional response to their work (Cammann et al., 1983) and numerous managerial coaching studies have examined the relationship between managerial coaching and employee satisfaction with work (Agarwal et al., 2009; Kalkavan and Katrinli, 2014; Ali et al., 2018; She et al., 2019). Managerial coaching behaviors including, listening to and offering encouragement to support employee professional and personal development (Ellinger et al., 2003), can result in employees feeling known, supported, and encouraged and are likely to increase an employees’ satisfaction with work (Kim et al., 2013). Similarly, coaching actions that demonstrate a manager’s interest in their employee’s development may lead to increased organizational commitment, or their employee’s connection, loyalty, and engagement with their organization (Meyer and Allen, 1997).

### 2.2 Path-goal leadership

Path–goal leadership theory guided this study (House, 1971; House and Mitchell, 1975; House, 1996), positing that managers can employ supportive behaviors to motivate their employees, improve job performance, and positively influence the study’s dependent variables of role clarity, job satisfaction, and organization commitment (House and Mitchell, 1975). As managers motivate and cultivate environments designed to improve employee performance through path–goal leadership behaviors (House, 1971), employees may become more committed to the organization, develop increased role clarity, and feel more satisfied with their jobs as their performance improves. Similarly, managerial coaching is often employed to help employees attain work goals through specific coaching behaviors such as clarifying expectations, removing obstacles, providing feedback, encouraging learning, and cultivating supportive work environments (Kumari et al., 2022). Managerial coaching behaviors parallel path-goal leadership behaviors described by House (1971, 1996) as both are intended to improve employee performance. As such, path–goal leadership serves a robust framework for exploring the influence of managerial coaching behaviors on workplace outcomes.

### 2.3 Measurement invariance

Best practices in statistical methodology assert that measurement invariance should be conducted prior to longitudinal analyses (Little, 2013). Testing for invariance occurs through a hierarchical sequence of tests within a SEM framework, with each additional test constraining further components of the model (Vandenberg and Lance, 2000). The first level of measurement invariance is configural invariance, which when found, suggests participants hold similar cognitive frames of reference or conceptual frameworks when responding to items across the data collection moments. The second level of invariance, or metric invariance, indicates factor loadings of items contribute to the latent construct equally across the data waves, suggesting the latent measure maintains a similar meaning and structure across time. Scalar invariance, the next (and final level for the present study), suggests that “measurement scales share the same operational definition” by testing whether the intercepts of indicators remain similar across time (Cheung and Lau, 2012, p. 168). Models are compared to one another after each level of testing to determine indications of invariance (Cheung and Rensvold, 2002).

Findings of measurement invariances in longitudinal study designs indicate that constructs are understood similarly across time lags (Putnick and Bornstein, 2016). However, few published studies have assessed measurement invariance within the empirical managerial coaching literature (Hammack-Brown, 2018). This number reduces further when considering only studies that utilized the CBI managerial scale (Ellinger et al., 2003). Pousa (2016) conducted measurement invariance between French and Spanish speakers using the CBI scale, finding support for invariance for six of the eight items, with two items showing non-invariance. Although no studies to date using the CBI instrument have tested for measurement invariance over time, given invariance findings with the CBI scale in cross-cultural settings (Pousa, 2016), the following hypothesis is proposed:

Hypothesis 1 (H1): There will be (a) configural, (b) metric invariance, and (c) scalar invariance from T1 and T2 between managerial coaching and each dependent variable (role clarity, job satisfaction, and organization commitment).

### 2.4 Cross-lagged design

Limited research exists to hypothesize cross-lagged differences regarding managerial coaching and role clarity over time, managerial coaching and job satisfaction over time, and managerial coaching and organization commitment over time. However, studies that conducted repeated measures and/or correlations of the hypothesized paths were consulted to inform hypotheses.

Substantial empirical research links managerial coaching with role clarity, highlighting the perceived ability of managerial coaching to enhance employee role clarity. In a study using structural equation modeling (SEM), Kim et al. (2013) found that managerial coaching had a standardized positive direct effect on role clarity of 0.42, resulting in a standardized total effect of 0.42. Moreover, Kim et al. (2014) in Study 1, reported that managerial coaching had a standardized positive direct effect on role clarity of 0.73, resulting in a standardized total effect of 0.73. In Study 2, Kim et al. (2014) found that managerial coaching had a standardized positive direct effect on role clarity of 0.48, resulting in a standardized total effect of 0.48. In the final identified study, Kim (2014) discovered that managerial coaching had a standardized positive direct effect on role clarity of 0.69, resulting in a standardized total effect of 0.69. Their findings infer that managerial coaching can positively influence role clarity, however, the cross-sectional research limits the ability to determine temporal precedence and examine the relationship over time. Despite these shortcomings, the identified studies illustrate evidence of a consistent positive relationship between role clarity and managerial coaching, signaling the potential for a positive relationship in a longitudinal study.

Similarly, the relationship between managerial coaching and job satisfaction is well documented in the literature, rooted in the theory that managerial behavior directly influences employee’s satisfaction levels (House, 1971). In demonstrating the relationship between job satisfaction and managerial coaching, Kim et al. (2013), reported a standardized positive direct effect of managerial coaching on job satisfaction of 0.43 and a standardized indirect effect through role clarity of 0.17, resulting in a standardized total effect of 0.60. Furthermore, in a study on U.S. public administration employees, Kim et al. (2014) discovered that managerial coaching had a standardized positive direct effect on job satisfaction of 0.30 and a standardized indirect effect through role clarity of 0.33, resulting in a standardized total effect of 0.63. Findings from these studies indicate a consistent replication of a cross-sectional relationship between the CBI managerial coaching scale and job satisfaction and provide rationale for exploring a longitudinal relationship. Although the studies reviewed above are limited by their cross-sectional data collection methods, the associations reported between job satisfaction and managerial coaching have been consistent, suggesting evidence of a positive relationship.

Numerous studies have also linked managerial coaching with organization commitment, demonstrating evidence that coaching can increase employees’ commitment to the organization. Kim et al. (2013) reported an observed correlation of 0.52 between managerial coaching and job satisfaction. Using SEM, they found that managerial coaching had a standardized indirect effect through job satisfaction of 0.46, resulting in a standardized total effect of 0.46. Discoveries from Kim et al. (2013) indicate a consistent replication of a cross-sectional relationship between managerial coaching and organization commitment. Similarly, Woo (2017), discovered a positive relationship (correlation of 0.29) between managerial coaching and organization commitment in their study on South Korean employees.

The overview of the empirically tested relationships between managerial coaching and the dependent variables of role clarity, job satisfaction, and organization commitment support positive associations between the variables of interest and indicate a consistent replication of a cross-sectional relationship. As a result, the second and third study hypotheses proposed are::

Hypothesis 2 (H2): There will be statistically significant positive cross-lagged effects between managerial coaching (T1) and each dependent variable (T2) (a) role clarity, (b) job satisfaction, and (c) organization commitment, controlling for the dependent variable (T1).

Hypothesis 3 (H3): The reciprocal cross-lagged effects between (a) role clarity (T1) and managerial coaching (T2), controlling for managerial coaching (T1), (b) job satisfaction (T1) and managerial coaching (T2), controlling for managerial coaching (T1), and (c) organization commitment (T1) and managerial coaching (T2), controlling for managerial coaching (T1), will be smaller than the cross-lagged effects from H2.

## 3 Methodology

### 3.1 Sample and procedures

Study participants were MBA students in the Southwest region of the US who work full-time in the US. All respondents were surveyed twice with a one-month time lag. The time lag between surveys was determined to be appropriate based on the belief that managerial coaching can cause change over a relatively short time period (Raza et al., 2018). Previous managerial coaching studies that have used time lags range from 1 month between data collection waves (Abid et al., 2020; Zhao and Liu, 2020; DuPlessis et al., 2021; Carrell et al., 2022) to 3 months (van Dierendonck and Dijkstra, 2012). Moreover, a 1 month time-lag enabled the researchers to effectively capture data collection with the time frame of a semester, which was a necessary requirement given the MBA student sample frame.

Invitations to participate in the survey were sent using the Qualtrics platform. Reminder emails were sent out three and 7 days after the initial survey invitation. Emails were sent at the beginning of each week and earlier in the day to align with online data collection best practices (Saleh and Bista, 2017). Screening questions were employed at the beginning of each survey to ensure data collected aligned with the study’s intended goals and research hypotheses (i.e., full-time, US employees). All participants indicated that there were no changes in supervisors/managers in the one-month time lag to ensure that any changes found in managerial coaching behavior across time would be related to the same supervisor. The survey also included an instructional manipulation check to confirm participants read instructions and items accurately.

A total of 313 participants completed both Survey 1 and Survey 2. Of the 313 respondents, 53% identified as women. In terms of age, 41% were aged between 18 and 34, whereas 59% were aged 35 and older. Additionally, 76% of respondents identified as non-Hispanic and 73% identified as Caucasian. After data collection concluded, chi-square tests were conducted to determine the presence of non-response bias across age, gender, race, and ethnicity. Results demonstrated no statistically (*p* ≤ 0.05) or practically significant differences (Cramer’s *V* ≥ 0.10) across gender, age, ethnicity, and race, indicating a lack of bias between the Survey 2 non-responders and the Survey 2 responders (Table 1).

**Table 1:** Non-response bias chi-square results.

	Survey 1 only (*n* = 314)	Survey 1 & 2 sample (*n* = 313)			
	% (*n*)	% (*n*)	χ^2^	*p*	Cramer’s *V*
Gender			2.4450	0.1180	0.062
Women	53 (185)	53 (165)			
Men	47 (129)	47 (148)			
Age			0.0850	0.7710	0.012
18–34	41 (258)	41 (127)			
35+	59 (369)	59 (186)			
Ethnicity			0.7860	0.3750	0.035
Not Hispanic	78 (488)	76 (239)			
Hispanic	22 (139)	24 (74)			
Race			1.6860	0.1940	0.054
Caucasian	69 (432)	73 (227)			
Other	25 (157)	23 (73)			

*df* = 1.

### 3.2 Measures

Managerial coaching was measured using eight statements, asking individuals to assess their managers’ coaching behaviors (Ellinger et al., 2003). A sample item is “My supervisor provides me with constructive feedback” (p. 444). Items were operationalized into 7-point Likert-type scales, with 7 meaning *almost always* and 1 meaning *almost never.* The role clarity scale from Rizzo et al. (1970) contained six statements on a 7-point Likert-type scale ranging from 7, meaning *very true*, to 1, meaning *very false*. Items asked participants about clear job expectations and guidelines. A sample statement is “I have clear, planned goals and objectives for my job” (p. 156). The job satisfaction scale, created by Cammann et al. (1983), included three items. A sample statement is “In general, I like working here” (p. 84). Responses were obtained using a 7-point Likert-type scale, where 7 indicates *strongly agree*, and 1 indicates *strongly disagree*. Organization commitment was measured using Meyer and Allen (1997) affective organization commitment subdimension. Each scale item operated on a 7-point Likert-type scale where 7 indicates *strongly agree*, and 1 indicates *strongly disagree*. A sample item is “I would be very happy to spend the rest of my career with this organization.” In addition to gathering data on the measures listed above, demographic information was collected. Demographic questions included gender, race, ethnicity, and tenure.

## 4 Analyses and results

Before testing the study hypotheses, a CFA for each model was conducted to verify the items from each measurement instrument appropriately loaded on their respective latent constructs. All models were estimated in SPSS ® Amos 28.0.0. Within each measurement model, items were constrained to load only on their respective factor, all latent constructs were allowed to correlate, and residuals were correlated across T1 and T2 (Little, 2013). A total of three measurement models were conducted, one for each dependent variable (i.e., role clarity, job satisfaction, and organization commitment). All measurement models indicated adequate fit based on the following goodness of fit criteria: χ^2^ likelihood ratio statistic, root mean square error of approximation (RMSEA), comparative fit index (CFI), Tuker-Lewis fit index (TLI), and the root mean square residuals standardized (SRMR) (Table 2). Since all factor loadings were significant and loaded above the recommended 0.50 threshold and less than 0.95 (Kline, 2016), all indicators were retained in each dependent variable model. Table 3 presents correlations among managerial coaching and the dependent variables.

**Table 2:** Measurement model fit indices for the dependent variable models.

Model	χ^2^	*df*	RMSEA	SRMR	TLI	CFI
Role clarity	554.786	330	0.047	0.053	0.963	0.968
Job satisfaction	311.206	192	0.045	0.032	0.976	0.980
Org. commitment	626.892	330	0.054	0.048	0.950	0.956

*df*, degrees of freedom; RMSEA, root mean square error of approximation; SRMR, standardized root mean square residual; CR, correlation residuals; TLI, Tucker-Lewis index; CFI, comparative fit index; Org., Organization.

**Table 3:** Implied and observed correlations, average variance extracted (AVE), and composite reliability (CR).

Model				
Role clarity (RC)	RC_T2	RC_T1	MC_T2	MC_T1
RC_T2	0.807	0.746	0.522	0.457
RC_T1	0.781	0.781	0.515	0.499
MC_T2	0.554	0.544	0.786	0.788
MC_T1	0.481	0.522	0.817	0.782
CR	0.917	0.902	0.927	0.926
AVE	0.651	0.610	0.617	0.612
Job satisfaction (JS)	JS_T2	JS_T1	MC_T2	MC_T1
JS_T2	0.841	0.100	0.550	0.508
JS_T1	0.899	0.886	0.550	0.508
MC_T2	0.640	0.596	0.787	0.788
MC_T1	0.553	0.550	0.817	0.770
CR	0.906	0.916	0.906	0.910
AVE	0.707	0.785	0.619	0.593
Org commitment (OC)	OC_T2	OC_T1	MC_T2	MC_T1
OC_T2	0.743	0.855	0.536	0.468
OC_T1	0.912	0.725	0.466	0.448
MC_T2	0.583	0.505	0.786	0.788
MC_T1	0.509	0.488	0.817	0.782
CR	0.879	0.866	0.927	0.926
AVE	0.552	0.525	0.617	0.612

Square root of AVE along the diagonal; T1 = Time 1; T2 = Time 2; observed correlations above the diagonal.

### 4.1 Measurement invariance

After confirmation of adequate fit for each dependent variable measurement model, measurement invariance was tested for each model, starting with using the CFA measurement model for each dependent variable. Testing for MI occurs through a hierarchical sequence of tests within a SEM framework, each additional test constraining further components of the model (Vandenberg and Lance, 2000). Models are then compared to one another after each level of testing to determine indications of invariance (Cheung and Rensvold, 2002). The entire MI sequence comprises up to four levels: configural, metric, scalar, and strict. However, the fourth level was not conducted in the study as it is considered overly stringent (Byrne, 2013). For each model, if invariance held given the commonly accepted fit indices of chi-square, RMSEA, and CFI, testing continued to the next nested level of invariance (metric and then scalar). A ∆CFI of 0.01 or less between the configural and metric and the metric and scalar levels demonstrated support for Hypothesis 1a, b, and c within each dependent variable model (Cheung and Rensvold, 2002).

Measurement invariance testing for the role clarity model started with the configural model. A review of the configural model fit indices demonstrated adequate fit with a chi-square of 554.786 (*df* = 330), a RMSEA of less than 0.08 (0.047), and a CFI of 0.968 (Table 4). Next the metric model was run, and fit indices were compared to the configural model. A CFI difference of less than 0.001 and a chi-square non-significant change at the 0.05 alpha level (*p* = 0.786) indicated metric invariance. The demonstration of metric invariance enabled testing to run the scalar model. Fit indices from the scalar model were compared to the metric model and demonstrated scalar invariance through a less than 0.001 change in CFI and a non-significant chi-square change (*p* = 0.328). Hypothesis 1 a, b, and c for the role clarity model were supported.

**Table 4:** Measurement invariance fit indices.

Model	χ^2^	*df*	RMSEA	CFI	ΔCFI	Δχ^2^	Δ*df*	*p*	Comparison
Role clarity									
Configural	554.786	330	0.047	0.968	-	-	-	-	
Metric	562.775	342	0.045	0.968	<0.001	7.989	12	0.786	Configural to metric
Scalar	576.357	354	0.045	0.968	<0.001	13.582	12	0.328	Metric to scalar
Job satisfaction									
Configural	311.206	192	0.045	0.980	-	-	-	-	
Metric	317.869	201	0.043	0.980	<0.001	6.663	9	0.672	Configural to metric
Scalar	322.853	210	0.042	0.981	0.001	4.984	9	0.836	Metric to scalar
Org commitment									
Configural	626.892	330	0.054	0.956	-	-	-	-	
Metric	636.667	342	0.053	0.956	<0.001	9.775	12	0.636	Configural to metric
Scalar	652.726	354	0.052	0.956	<0.001	16.059	12	0.189	Metric to scalar

RMSEA, root mean square error of approximation; CFI, comparative fit index.

Testing measurement invariance for the job satisfaction model began with the configural level. The configural model depicted adequate model fit (χ^2^ = 311.206 (*df* = 192), CFI = 0.980, RMSEA = 0.045; Table 4), which provided support for Hypothesis 1a and therefore testing continued to metric invariance. A less than 0.001 CFI difference between the metric and configural model and a non-statistically significant difference in chi-square (*p* = 0.672) provided ample support for metric invariance and Hypothesis 1b. Testing then continued to the last nested model of invariance testing, scalar invariance. The model was run and fit indices were compared. With a CFI difference between models of 0.001 and a non-statistically different chi-square between the scalar and metric model (*p* = 0.836), scalar invariance between managerial coaching and job satisfaction over time was supported (Hypothesis 1c).

The organization commitment model configural model fit indices were first reviewed. A χ^2^ of 626.892 (*df* = 330), a CFI of 0.956, and a RMSEA of 0.054 (Table 4) demonstrated adequate fit therefore Hypothesis 1a was supported and further testing proceeded to metric invariance. The metric model was then compared to the configural model, which revealed a change in CFI of less than 0.001 and a non-statistically significant difference in chi-square values (*p* = 0.636), suggesting metric invariance and providing support for Hypothesis 1b. Testing for scalar invariance was then conducted. A comparison of the metric to the scalar invariance models demonstrated a ΔCFI of less than 0.001 and a non-statistically significant *p*-value (*p* = 0.189). Results indicated support for Hypothesis 1 a, b, and c.

### 4.2 Cross-lagged panel modeling and reciprocal cross-lagged effects

Data analysis then turned to cross-lagged panel modeling (CLPM) to test Hypotheses 2 and 3. Three CLPMs were built based on the respective scalar invariance CFA model; each model focused on a different dependent variable. All three models specified autoregressive paths from managerial coaching at T1 to managerial coaching at T2 and the dependent variable at T1 to the dependent variable at T2. Additionally, cross-lagged paths were specified from managerial coaching at T1 to the outcome at T2 and vice versa. Residual variances (i.e., errors) from each indicator were correlated across time, and latent variables at T1 and T2 were correlated with each other. Additionally, since each CLPM was based on the scalar invariance model, factor loadings and intercepts of paralleled constructs were still constrained to be equal. Partial $\eta^{2}$ was also calculated to determine the practical significance of the results. Results for each model are specified below.

Fit indices for the role clarity CLPM encompassed a χ^2^ of 576.357 (*df* = 354), a 0.968 CFI, and a RMSEA of 0.045 thereby indicating good fit. The beta weight between managerial coaching T1 and role clarity T2, controlling for role clarity T1, was 0.104 and statistically significant (*p* = 0.027, η_p_^2^ = 0.021). Results indicated support for Hypothesis 2a. However, a review of the reciprocal cross-lagged coefficient did not support Hypothesis 3a. The reciprocal cross-lagged coefficient between role clarity T1 and managerial coaching T2, controlling for managerial coaching T1, was 0.160 and also statistically significant (*p* = 0.011, η_p_^2^ = 0.057). Calculations revealed a higher reciprocal cross-lagged effect than the cross-lagged beta weight of 0.104 (*p* = 0.027).

Fit indices for the job satisfaction CLPM revealed a χ^2^ of 322.853 (*df* = 210), a 0.981 CFI, and a RMSEA of 0.042, illustrating adequate fit. The standardized coefficient between managerial coaching at T1 and job satisfaction at T2, controlling for job satisfaction at T1, was not statistically significant (0.084, *p* = 0.055, η_p_^2^ = 0.026). The lower bound confidence interval for the cross-lagged coefficient was less than zero (−0.003), providing additional evidence of a non-statistically significant effect. Therefore, Hypothesis 2b was not supported. The reciprocal cross-lagged coefficient between job satisfaction at T1 and managerial coaching at T2, controlling for managerial coaching at T1, resulted in a statistically significant and higher coefficient (0.209, *p* = 0.004, η_p_^2^ = 0.090) than the cross-lagged coefficient from Hypothesis 2b of 0.084 (*p* = 0.055). A difference of 0.125 was found between the two cross-lagged beta weights. Due to the lack of supporting evidence, Hypothesis 3b was also not supported.

Within the organization commitment CLPM, fit indices were identical to the scalar invariance model with a χ^2^ of 652.726 (*df* = 354), a CFI of 0.956, and a RMSEA of 0.052; all indices indicating good fit. The standardized coefficient between managerial coaching at T1 and organization commitment at T2, controlling for organization commitment at T1, was 0.086 and statistically significant at *p* = 0.036, providing evidence to support Hypothesis 2c. However, the reciprocal cross-lagged coefficient between organization commitment T1 and managerial coaching T2, controlling for managerial coaching T1, was 0.139 (*p* = 0.016, η_p_^2^ = 0.045), which was a higher coefficient than the cross-lagged coefficient. Therefore, Hypothesis 3c was not supported. See Table 5 for a summary of findings.

**Table 5:** Results summary of predicted hypotheses.

Hypotheses	Supported?	Notes
H1. Measurement invariance	Yes	a. All models adequate configural fit b. All models configural to metric ΔCFI of <0.001; c. RC: metric to scalar ΔCFI of <0.001; JS: ΔCFI of 0.001; OC: ΔCFI of <0.001;
H2. Cross-lagged effect	Partially; JS (3b) not supported	a. RC_T2 < -- MC_T1:β of 0.104 (*p* = 0.027); η_p_^2^ = 0.021 b. JS_T2 < -- MC_T1: β of 0.084 (*p* = 0.055); η_p_^2^ = 0.026 c. OC_T2 < -- MC_T1: β of 0.086 (*p* = 0.036); η_p_^2^ = 0.035
H3. Reciprocal cross-lagged effect	No	a. MC_T2 < --RC_T1: β of 0.160 (*p* = 0.011); η_p_^2^ = 0.057 b. MC_T2 < --JS_T1: β of 0.209 (*p* = 0.004); η_p_^2^ = 0.090 c. MC_T2 < --OC_T1: β of 0.139 (*p* = 0.016); η_p_^2^ = 0.045

RC, role clarity model; JS, job satisfaction model; OC, organization commitment model.

## 5 Discussion of findings

This study sought to assess the predictive validity of the Employee Perceptions of Supervisor/Line Manager Coaching Behavior Measure (CBI) by determining measurement invariance and then conducting a cross-lagged panel design. The findings attest to the predictive value of managerial coaching in the workplace, but simultaneously call into question the assumed one-way directional relationship between managerial coaching and workplace attitudes and behaviors such as role clarity, job satisfaction, and organization commitment. Discussions, implications, and future research are presented in the sections that follow.

### 5.1 Measurement invariance

Study findings supported configural, metric, and scalar invariance within each dependent variable model, demonstrating that the relationships between the variables remained consistent over time (Millsap and Cham, 2012). The present study built upon previous research by Pousa (2016), who conducted measurement invariance of the CBI scale between French and Spanish speakers. Pousa (2016) discovered metric invariance across the two language groups for all but two of the eight scale items, confirming partial MI of the CBI scale items. The present study’s findings expand knowledge by demonstrating full metric and scalar invariance across all eight CBI scale items as well as across all role clarity, job satisfaction, and organization commitment scale items. Demonstrating measurement invariance of the CBI scale has implications for researchers, specifically for those who desire to conduct future managerial coaching longitudinal studies as construct consistency remains a prerequisite to appropriately and adequately explain change over time.

### 5.2 Cross-lagged panel models

Study analyses also included three cross-lagged panel models to determine the predictive validity of the CBI scale with each dependent variable. The role clarity model contained a positive direct effect between managerial coaching at T1 and role clarity at T2 while controlling for role clarity at T1, illustrating that managerial coaching behaviors directly affected employee role clarity across a 1-month time lag, above and beyond previous employee role clarity levels. The $\eta_{p}^{2}$ indicated that approximately 2% of the variance in role clarity at T2 that is not common with role clarity at T1 can be explained by managerial coaching at T1. The organization commitment model led to a similar finding with a statistically significant cross-lagged effect from managerial coaching at T1 to organization commitment at T2, controlling for organization commitment at T1. Results were further clarified by $\eta_{p}^{2}$ , which demonstrated managerial coaching at T1 explained approximately 4% of the variance in organization commitment at T2 that is not common with organizational commitment at T1. Taken together, the results provide convincing evidence of a cross-lagged association between managerial coaching and role clarity and managerial and organization commitment, demonstrating that managers who engage in managerial coaching can foster increased role clarity and organization commitment over time in those they coach.

Results from the CLPM job satisfaction model differed significantly from the aforementioned two models with a non-statistically significant effect of 0.084 (*p* = 0.055; $\eta_{p}^{2}$ = 0.026) from managerial coaching at T1 to job satisfaction at T2 while controlling for job satisfaction at T1. The non-statistically significant cross-lagged effect suggests that managerial coaching behaviors did not predict future levels of job satisfaction when adjusting for prior satisfaction levels. This result was unexpected, given statistically significant effects in prior cross-sectional studies (Kim et al., 2013; Kim, 2014). The high job satisfaction autoregressive effect of 0.852 (*p* = 0.002) between T1 and T2 indicates that little change in overall job satisfaction levels occurred during the month between data collection intervals and may explain the surprising finding. Since the job satisfaction stability coefficient accounted for a large portion of the job satisfaction variance, the cross-lagged effect did not have much change to explain, which might have resulted in underestimating the effect (Little, 2013).

Another striking finding concerns the relatively similar stability coefficients in the role clarity model, intimating a reciprocal relationship between role clarity and managerial coaching which suggests the variables mutually influence each other. Kenny and Harackiewicz (1979) argued that researchers should only consider two-way causation, or reciprocal relationships, when variable stabilities are nearly equal as “cross-lagged difference is affected by the relative stability of both variables” (p. 377). The nearly identical stability coefficients of 0.734 (*p* < 0.001) for managerial coaching and 0.727 (*p* = 0.002) for role clarity suggest no potential bias in comparing the two beta weights. This finding may seem counter-intuitive to current managerial coaching understanding, which posits managerial coaching leads to increased role clarity and not the other way around. Current managerial coaching literature provides a limited understanding of managerial coaching and role clarity temporal dynamics because understanding rests predominately in cross-sectional research, which cannot inform effect directionality. This finding suggests that employee attitudes may impact perceived leader behaviors in addition to leadership behaviors impacting employee attitudes. A possible interpretation is that employees with high role clarity may be more aware of managerial coaching behaviors from their managers, given their understanding of their role and expectations. Or perhaps employees who possess a clear understanding of their role and expectations are easier for managers to coach or are more eager to be coached. These indications are confirmed by recent studies examining employee coachability (Weiss and Merrigan, 2021), citing that employee disposition plays a pivotal role in the effectiveness of managerial coaching.

Also noteworthy are the relatively high managerial coaching stability coefficients between T1 and T2 for managerial coaching in the role clarity model (0.734, *p* < 0.001) and in the organization commitment model (0.749, *p* < 0.001). The high stability coefficients indicate that relatively little change occurred in perceived managerial coaching behaviors across 1 month, confirming prior studies that have demonstrated the relative stability of leadership behaviors over time (Volmer et al., 2011; Brown and Chai, 2012). However, the high managerial coaching autoregressive effect is an interesting finding as managerial coaching stability was unresearched prior to this study. Although past literature has indicated that leadership behaviors remain stable over time (Volmer et al., 2011; Brown and Chai, 2012), the discovery of a high stability coefficient for managerial coaching over a 1-month time lag is a unique contribution to the field. Given calls in the literature to conduct managerial coaching longitudinal studies, evidence regarding the stability of managerial coaching demonstrates sound psychometric properties of the CBI scale.

### 5.3 Reciprocal cross-lagged effects

The reciprocal cross-lagged effect between each dependent variable at T1 and managerial coaching at T2 while controlling for managerial coaching at T1 were examined. Contrary to our hypotheses, the reciprocal cross-lagged coefficient in each model was higher than the cross-lagged coefficient across all three models. The reciprocal cross-lagged effect between role clarity at T1 and managerial coaching at T2, controlling for managerial coaching at T1, was statistically significant, proffering a difference of 0.056 between the coefficients. The reciprocal cross-lagged coefficient between job satisfaction at T1 and managerial coaching at T2, controlling for managerial coaching at T1, resulted in a statistically significant and higher coefficient, presenting a difference of 0.125 between the two cross-lagged beta weights. Concerning the organization commitment model, a difference of 0.053 between the cross-lagged and reciprocal cross-lagged coefficient was found. The reciprocal cross-lagged effects demonstrate that employee attitudes such as role clarity, organization commitment, and job satisfaction were related to increased managerial coaching behaviors 1 month later, or more broadly, that employee attitudes may positively influence perceived manager’s coaching behaviors over time.

These results may appear illogical compared to current perspectives on managerial coaching, which posit that managerial coaching leads to increased employee outcomes and not the other way around. Extant research provides a limited understanding of managerial coaching and employee outcome temporal dynamics as understanding rests predominately in cross-sectional research. The higher reciprocal cross-lagged effects support the idea of a reciprocal relationship, suggesting that employee attitudes may impact perceived leader behaviors in addition to leadership behaviors impacting employee attitudes. The study’s design to include the reciprocal cross-lagged effects highlighted a new insight regarding the influence of employee attitudes on perceived manager’s coaching behaviors. Results suggest that role clarity, organization commitment, and job satisfaction may nurture and influence managerial coaching behaviors.

### 5.4 Implications for practice and theory

A number of implications for practitioners are offered based upon the findings of this study. Although previous cross-sectional research posits a positive relationship between managerial coaching and job satisfaction (Kim et al., 2013; Kim, 2014; Zhao and Liu, 2020), current study findings did not support the notion that managerial coaching predicts change in job satisfaction within a 1-month time frame. Therefore, managers are cautioned against relying solely on managerial coaching as a mechanism to increase employee job satisfaction, as it may not be as effective as emphasized in the current literature.

Additionally, organizational leaders and managers who hope to increase managerial coaching behaviors should consider the role of workplace attitudes, such as role clarity, satisfaction, and commitment, as a means to support internal organizational development tools and strategies. Recent studies have noted that highly coachable individuals remain critical to the success of managerial coaching in organizations (Weiss and Merrigan, 2021). Study findings support the concept that the happier and more committed employees are to the organization and the clearer they understand their role, the more employees may perceive the managerial coaching behaviors of their manager. Therefore, while some emphasis should remain on developing the manager with the training, skills, and behaviors to deploy managerial coaching effectively, organizational leaders should also consider employee attitudes, behaviors, and coachability when implementing coaching and developing training.

In terms of theoretical implications, calls in the literature have emphasized the necessity for more robust connections between managerial coaching and leadership theories (Hamlin et al., 2008; Ellinger et al., 2011; Anderson, 2013). The present study, theoretically grounded in path-goal leadership (House, 1971), drew upon this leadership theory to elucidate how managerial coaching can lead to workplace outcomes such as role clarity, satisfaction with work, and organization commitment over time. The role of time between managerial coaching and workplace behaviors and outcomes has not been addressed within the path-goal leadership framework until this study, adding a notable contribution to path-goal leadership theory development. However, the higher reciprocal cross-lagged effects found in the current study are not fully explained by path-goal leadership theory, which posits that leadership behavior generates and motivates change in employee workplace outcomes (House, 1971). Findings from the study allude to the necessity for a new theory to explicate these unique results or a reevaluation of the path-goal leadership concept. Further theoretical understanding is necessary to comprehend the relationships between managerial coaching, role clarity, job satisfaction, and organization commitment, specifically to explain the high reciprocal cross-lagged effects.

Taking these perspectives together, results indicate to practitioners and researchers that coaching remains an effective practice to increase employee role clarity and organization commitment. Unique is the finding that these workplace outcomes increase over a one-month time span. Therefore, practitioners should continue developing and enhancing training and organizational development initiatives targeted at developing managerial coaching skills in managers. Furthermore, and perhaps more notably, organizations should consider employee coachability, attitudes, and behaviors when engaging and implementing managerial coaching, finding opportunities to empower those who are coached. Additionally, the study’s theoretical implications indicate that a deeper theoretical understanding is essential to unravel the intricate connections between the study variables beyond path-goal leadership. Given the changing business landscape that calls upon managers and businesses to do more with less, such strategies may result in more positive workplace behaviors, attitudes, and outcomes.

### 5.5 Limitations and future research

A primary limitation lies with the study design choice. Although cross-lagged panel models offer a more robust method of understanding causal relationships over time, results stemming from these types of panels are limited in how they can explain change. The autoregressive and cross-lagged effects within the model depict individual differences in change; however, they are restricted from describing intraindividual change (Selig and Little, 2012). An additional limitation concerns the method of implementing managerial coaching. The survey was deployed during the COVID-19 pandemic, at the peak of returning back to face-to-face work environments. The study design did not inquire how the coaching was employed between manager and employee (i.e., face-to-face or virtual), nor did it ask if the deployment method changed during the 1-month time lag. Although previous studies have indicated the difference in virtual and non-virtual environments does not affect managerial coaching (Hammack-Brown, 2018), not accounting for these differences in coaching deployment methods is a limitation of the current study. Another limitation concerns the use of 1 month as a time lag as it is possible that changes in employees’ perceptions of their job satisfaction may require a longer period of time which may have influenced our findings. A final limitation is that results may not be generalizable to the intended population. The chi-square analysis comparing sample demographics to the general US full-time worker population revealed statistically significant and practically significant results across gender, age, race, and ethnicity.

Despite these limitations, the study offers numerous avenues for further research. A beneficial line of future research would be researching, producing, and verifying time lag guidelines. As the number and scope of longitudinal studies grow, additional research in this area would fill a current literature gap and support researchers as they pursue robust survey and statistical methodologies. Within managerial coaching research, future research could explore the influence of additional manager and employee demographics related to their levels, along with industry sectors, and any training on coaching that may have been received. In addition, gender, tenure, and coaching deployment methods on the relationships between managerial coaching and role clarity, job satisfaction, and organization commitment should be examined over time. Previous research has indicated that gender, tenures, and coaching deployment methods (i.e., virtual vs. face-to-face) may impact the coaching relationship as well as workplace outcomes such as job satisfaction and organization commitment (Ellinger et al., 2011; Ye et al., 2016; Woo, 2017; Hammack-Brown, 2018). These factors within a coaching relationship were not accounted for in the study and may be worth additional exploration. Results also highlight a need for additional research to understand the non-statistically significant cross-lagged effect between managerial coaching at T1 and job satisfaction at T2 while controlling for job satisfaction at T1. It is possible that different time lags (such as 3-month and 6-month time-lags) could capture changes in job satisfaction due to managerial coaching. With the strong evidence in previous studies regarding a positive relationship between managerial coaching and job satisfaction, additional research is warranted to yield supplemental insights.

In conclusion, the findings from this study illuminate new insights and understanding regarding measurement invariance, the psychometric properties of the CBI managerial coaching scale, and the reciprocal influence of employee outcomes on coaching behaviors. The longitudinal design of the current study expands knowledge concerning the relationship of change between managerial coaching and workplace outcomes over time. Findings support the notion that managerial coaching can be used as an impactful developmental tool for managers seeking to develop and support their employees, specifically related to role clarity and organization commitment. Moreover, results indicate change can occur in as little as 1 month. Additionally, results demonstrate the complexity of the relationships between managerial coaching and the dependent variables of role clarity, job satisfaction, and organization commitment. The reciprocal cross-lagged effect was larger than the cross-lagged effect in every dependent variable model, which challenges the hypothesized theoretical direction previously validated in cross-sectional studies (Kim et al., 2013; Kim, 2014). This finding also intimates that the relationships between managerial coaching and the dependent variables are mutually influential, meaning employee attitudes and behaviors may also affect managerial coaching behaviors. Role clarity, job satisfaction, and organizational commitment have been linked to positive organizational outcomes such as decreased turnover and increased performance (Kim et al., 2013; Abid et al., 2020). Therefore, managerial coaching remains a valuable workplace development tool for today’s business needs and challenges, especially where retention issues or employee commitment may be a concern (Kim, 2014; Woo, 2017; Abid et al., 2020).

## Data availability statement

The raw data supporting the conclusions of this article will be made available by the authors, without undue reservation.

## Ethics statement

The studies involving humans were approved by UT Tyler Human Research Protections Program (HRPP). The studies were conducted in accordance with the local legislation and institutional requirements. The participants provided their written informed consent to participate in this study.

## Author contributions

KS: Writing – original draft, Writing – review & editing. KN: Supervision, Writing – review & editing. AE: Writing – review & editing.
