# Supplementary material for: Examining the predictive validity of a managerial coaching scale: a longitudinal study
Source: Front Psychol. 2024 Apr 2;15:1277422. doi: 10.3389/fpsyg.2024.1277422 (PMC11020080; doi:10.3389/fpsyg.2024.1277422)
Supplement: Supplementary file 1 [file Image_1.pdf]

Supplementary Material

1 Supplementary Figures and Tables

Role Clarity (RC) Model

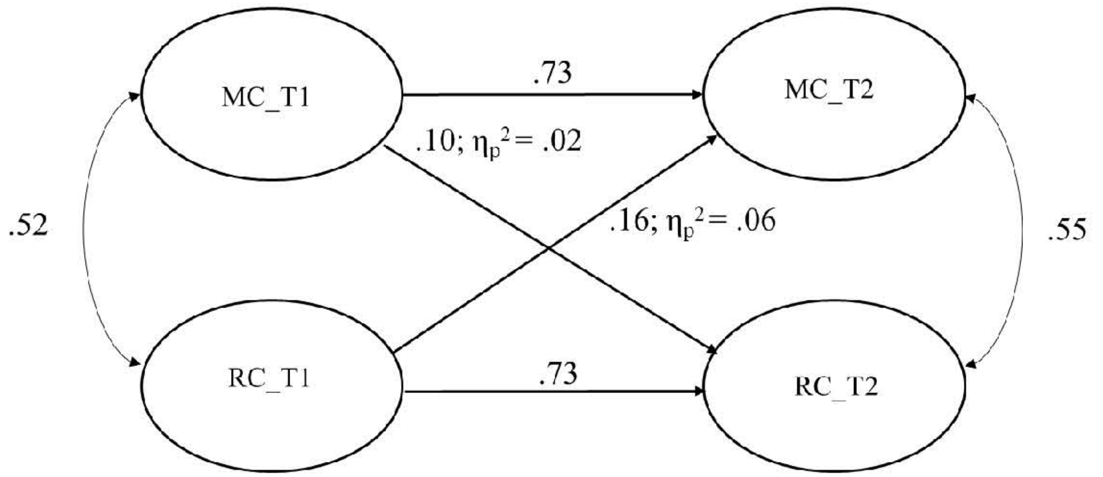

Job Satisfaction (JS) Model

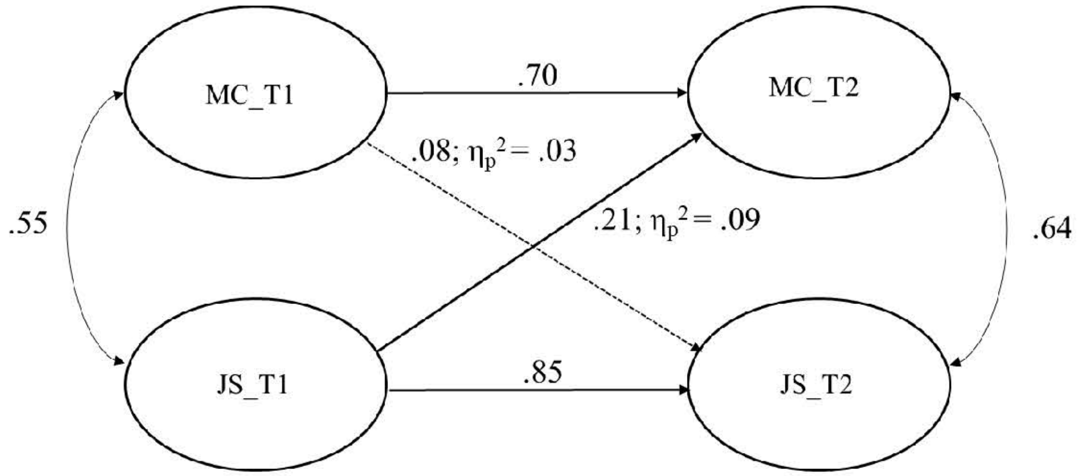

Organization Commitment (OC) Model

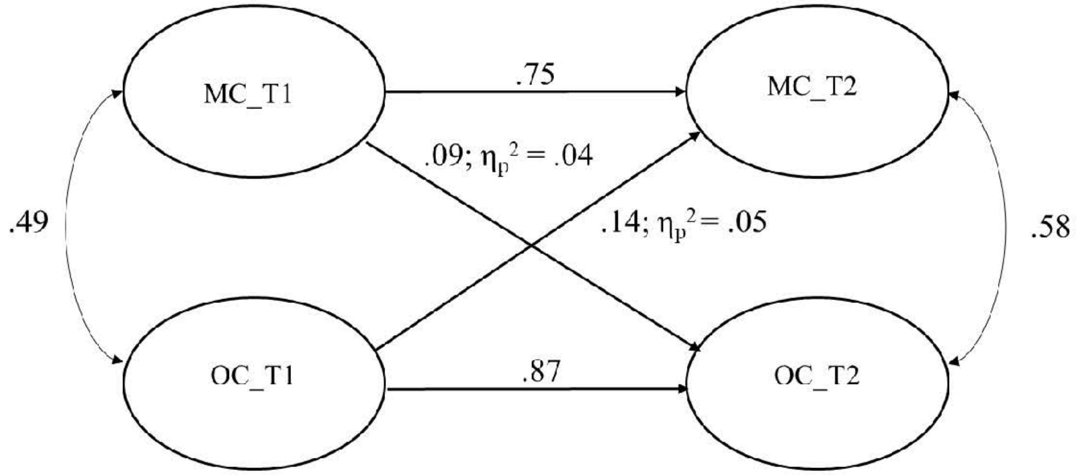

Supplementary Figure 1. Longitudinal Cross-Lagged Models
